# A novel thinking: DDR axis refines the classification of ccRCC with distinctive prognosis, multi omics landscape and management strategy

**DOI:** 10.3389/fpubh.2022.1029509

**Published:** 2022-11-21

**Authors:** Aimin Jiang, Jiaao Song, Xiao Fang, Yu Fang, Zheng Wang, Bing Liu, Zhenjie Wu, Le Qu, Peng Luo, Linhui Wang

**Affiliations:** ^1^Department of Urology, Changhai Hospital, Navel Medical University (Second Military Medical University), Shanghai, China; ^2^Department of Urology, Changzheng Hospital, Naval Medical University (Second Military Medical University), Shanghai, China; ^3^Department of Urology, The Third Affiliated Hospital, Naval Medical University (Second Military Medical University), Shanghai, China; ^4^Department of Urology, Affiliated Jinling Hospital, Medical School of Nanjing University, Nanjing, China; ^5^Department of Oncology, Zhujiang Hospital, Southern Medical University, Guangzhou, China

**Keywords:** renal cell carcinoma, DNA damage response, molecular subtypes, multi-omics, DDX1

## Abstract

**Background:**

DNA damage response and repair (DDR) related signatures play an important role in maintaining genome stability and other biological processes. It also affects the occurrence, development, and treatment of cancer. However, in renal cell carcinoma (RCC), especially clear cell renal carcinoma (ccRCC), the potential association between DDR-related signatures and tumor heterogeneity and tumor microenvironment (TME) remains unclear.

**Methods:**

Utilizing unsupervised clustering algorithm, we divided RCC into two subgroups, DCS1 and DCS2, according to the differences in DDR gene expression, and compared the characteristics of the two subgroups through multiple dimensions.

**Results:**

Compared with DCS1, DCS2 patients have higher clinical stage/grade and worse prognosis, which may be related to active metabolic status and immunosuppression status. At the same time, the high mutation rate in DCS2 may also be an important reason for the prognosis. We also analyzed the sensitivity of the two subgroups to different therapeutic agents and established a subtypes' biomarkers-based prognostic system with good validation results to provide ideas for clinical diagnosis and treatment. Finally, we identified a pivotal role for DDX1 in the DDR gene set, which may serve as a future therapeutic target.

**Conclusion:**

This study showed that DDR has an important impact on the development and treatment of RCC. DCS2 subtypes have a poor prognosis, and more personalized treatment and follow-up programs may be needed. The assessment of DDR gene mutations in patients may be helpful for clinical decision-making. DDX1 may be one of the effective targets for RCC treatment in the future.

## Introduction

Renal cell carcinoma (RCC) caused by genetic alterations accounts for approximately 2% of all adult carcinoma (1), and is the second leading cause of death associated with urologic malignancies (2). Clear cell renal cell carcinoma (ccRCC) leads the most common histological type of renal cancer, accounting for ~75% of renal cell carcinomas, ccRCC has a higher invasive capacity and recurrence rate than other renal cell carcinoma subtypes. The morbidity and mortality of ccRCC has been increasing rapidly in the last decades. Overall patient survival is not satisfactory (3) because of local recurrence and distant metastasis. Despite the effectiveness of targeted therapies and immunotherapy in the treatment of ccRCC, only some patients have achieved drug responsiveness, and most patients have intrinsic resistance or will eventually develop acquired resistance. Therefore, the identification of novel biomarkers and therapeutic agents is important for the clinical management of ccRCC patients.

The DNA damage response (DDR) is a highly conserved genomic monitoring mechanism that is activated when DNA damage occurs and functions accordingly to maintain cellular integrity and stability (4). DDR not only is involved in maintaining genomic integrity and cell viability, but also plays a critical role in some of the most commonly used anti-cancer therapies, such as targeting DNA (5). Cytotoxic agents targeting DDR pathways have been used as anti-cancer therapies. Some DDR kinase inhibitors have been reported to have progressed to clinical trials (6–8). These include kinase inhibitors for ATM, ATR and PLK1. Recently, the combination of DDR and tumor immunity has become a new hotspot (9), and relevant clinical trials are being carried out (10). However, the role of DDR in the progression and metastasis of ccRCC is unclear.

In this study, we performed a remodeling analysis based on DDR related signatures in ccRCC, and the subtypes were identified and verified across different datasets and compared at multi omics level. We decoded the heterogeneity and crosstalk of DDR and immune infiltration, genomic instability, drug therapy sensitivity *via* multi algorithms and datasets. All the findings retrieved from this work might be valuable for precise management and risk stratification of ccRCC patients.

## Materials and methods

### Data collection and processing

Multi omics datasets, including expression, genomic mutation, copy number variation, DNA methylation profiles, were retracted form UCSC Xena datasets (including ccRCC cohort, including 526 tumor and 70 normal samples) (11). Out-house datasets of ccRCC, including gene expression and clinical information of the Japan renal cancer cohort, Motzer's cohort and Wuttig's cohort, were download form public datasets (Access numbers were as follow: E-MTAB-1980, EGAS00001004353, GSE55241), containing nearly 1000 ccRCC tumor sample clinical and transcriptome information (12, 13). In addition, we also applied several online datasets, including MEXPRESS, UALCAN and TIDE, to validate results found in our study (14–16). For datasets collected from public cohorts, the informed consent or instructional review board approval were not required.

### Identification of different DDR subclusters in CcRCC

Altogether, we collected and filtered DDR related signatures from previous research and several datasets, including CPDB, KEGG, Reactome and MSigDB. The detailed DDR signatures were summarized in Supplementary Table S1. Based on expression profile of the DDR related signatures, we performed unsupervised consensus cluster analysis by R package “ConsensusClusterPlus.” Totally, patients from TCGA-KIRC cohort were sub-grouped into two distinct phenotypes, and k = 2 was identified as the optimal cluster number.

### Enrichment analysis between subgroups

Based on cluster results, we next deciphered the inner heterogeneity between subtypes. We firstly calculated differentially expressed genes (DEG) *via* R package “DEseq2” (The threshold was as follow: *p*-value < 0.01, and the abstract log Foldchange >2). After identifying DEG, we utilized R package “ClusterProfiler” to carry on annotation analysis, including Gene Ontology (GO), Kyoto Encyclopedia of Genes and Genomes (KEGG) pathways Gene Set Variation Analysis (GEVA) and Gene Set Enrichment Analysis (GSEA). The annotation files for DEG were downloaded from MSigDB and ConsensusPathDB (17).

### Different landscape of immune infiltration signatures

We applied several mainstream and robust immune related algorithms to calculate immune infiltration degree, cellular components, or immune cell enrichment scores between subtypes. In addition, single-sample gene set enrichment analysis (ssGSVA) was applied to prove the differences of immune heterogeneity between DCS1 and DCS2 (18). R package “ESTIMATE” was used to evaluate the stromal and immune scores in tumor microenvironment. Tumor Immune Dysfunction and Exclusion (TIDE) algorithm (16) was introduced to estimate immunotherapy responses.

### Mutation spectrum characteristics between subgroups

We downloaded mutation profiles of ccRCC, then compared and visualized the difference between DCS1 and DCS2 through R package “Maftools” (19). Besides, the oncogenic pathway and mutually exclusive or coexisting mutations were analyzed through function form “Maftools” (20). Analysis of loss and gain in genomic level was performed by GISTIC 2.0 algorithm (21).

### Drug sensitivity prediction

Through expression profile, we calculated each patient's therapy sensitivity through Genomics of Cancer Drug Sensitivity (GDSC) database, containing cancer cell lines transcriptome and molecular agents' response information. We estimated the half-maximal inhibitory concentration-IC50 and validated such difference by R package “pRRophetic.” Furthermore, we utilized two public and comprehensive datasets, CellMiner (22) and CCLE (23) to verify and identify novel treatment agents for ccRCC patients.

### Construction of risk prediction model

We firstly identified each subtype's biomarkers and filter signatures correlated with overall survival outcome by Cox analysis. Then, we ranked the importance of signatures on patients' prognosis *via* random forest algorithm and identified the optimal combination by Random Survival Forest Variable Hunting (RSFVH) algorithm. Patients in training and test cohorts were divided into high- and low- risk subtypes according to median risk score of each cohort.

### Statistical analysis

All omics dataset's processing, visualization and statistical analysis were finished by R software (version 4.1.3). For quantitative variables, Kruskal-Wallis and *t*-test were applied; as for qualitative characteristics, Chi-square was employed to compare the difference. Correlation among variables were based on R package “corrplot.” R packages “survival” and “pROC” were used to plot Kaplan-Meier and time ROC curves. All two-sided *p*-value (< 0.05) was considered statistically significant.

## Results

### Identification of subtypes *via* DDR-related signatures' profile

**Figure 1:**
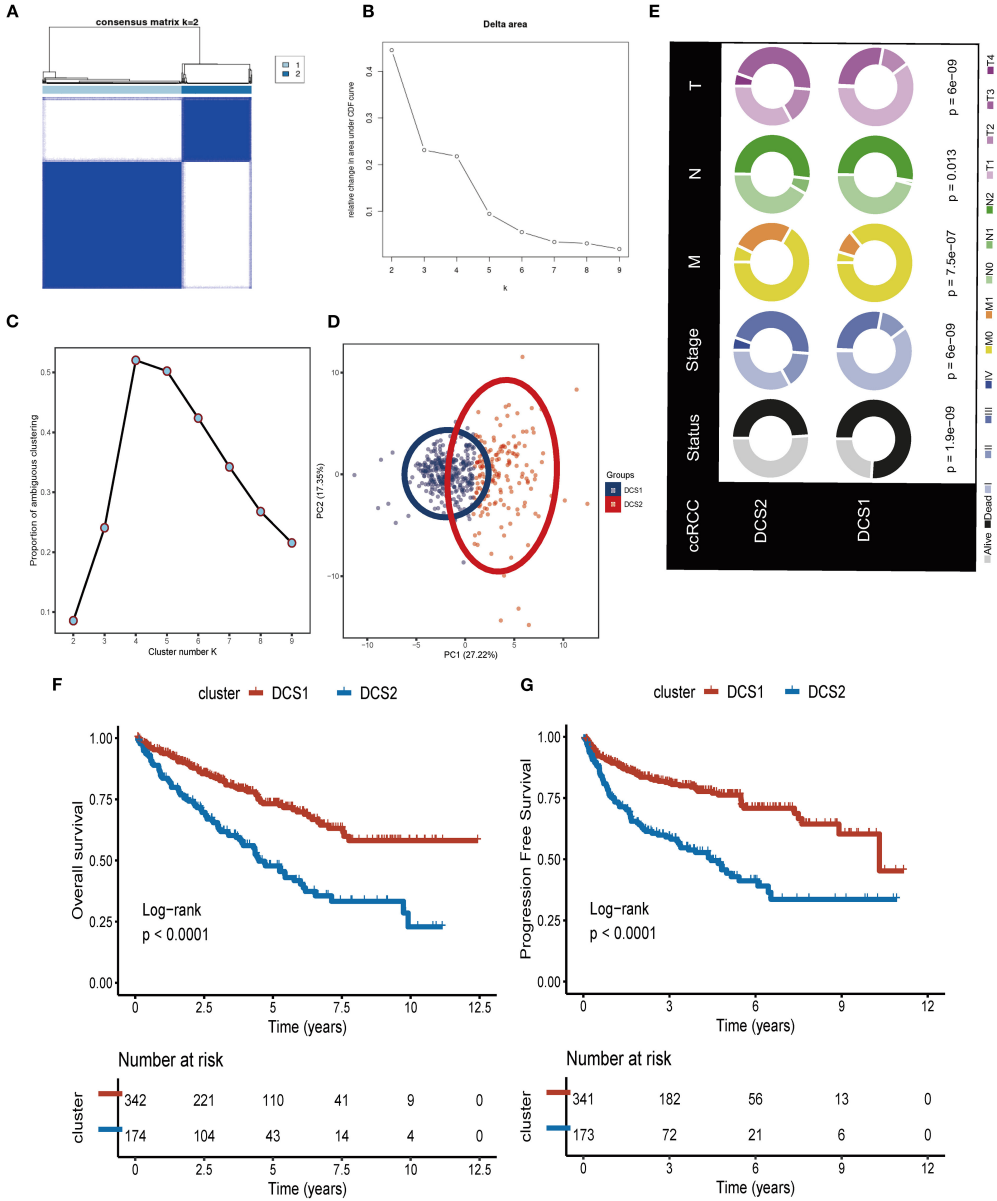
Identification of two DDR related subtypes. **(A)** Consensus cluster matrix based on DDR-related regulators. **(B)** Relative change in area under cumulative distribution function (CDF) curve. **(C)** The proportion of ambiguous clustering score, and the optimal cluster number. **(D)** Two-dimensional principal component plot based on DDR related regulators. **(E)** Clinical difference between DCS1 and DCS2. **(F,G)** Survival analysis of OS and PFS.

We firstly identified DDR regulators impacting on ccRCC patients' prognosis *via* Cox. Then, based on the expression level of those DDR regulators, unsupervised clustering was introduced to categorize the TCGA-ccRCC samples into different molecular subtypes. As shown in Figures 1A–D, we classified ccRCC into two clusters: DDR-associated cancer subtype 1 (DCS1) and DCS2. The clinical significance of this clustering approach was assessed by comparing the clinical outcomes of the two subtypes (Figure 1E). The results showed that patients in DCS1 had a better survival outcome (Figures 1F,G). The detailed clinical characteristic difference was summarized in Supplementary Table S2. In addition, we found that most DDR-related signatures were significantly upregulated in DCS2 (Supplementary Figure S2), suggesting that abnormal DNA damage repair signature is associated with a worse tumor prognosis.

### Functional enrichment analysis of different DDR subtypes

**Figure 2:**
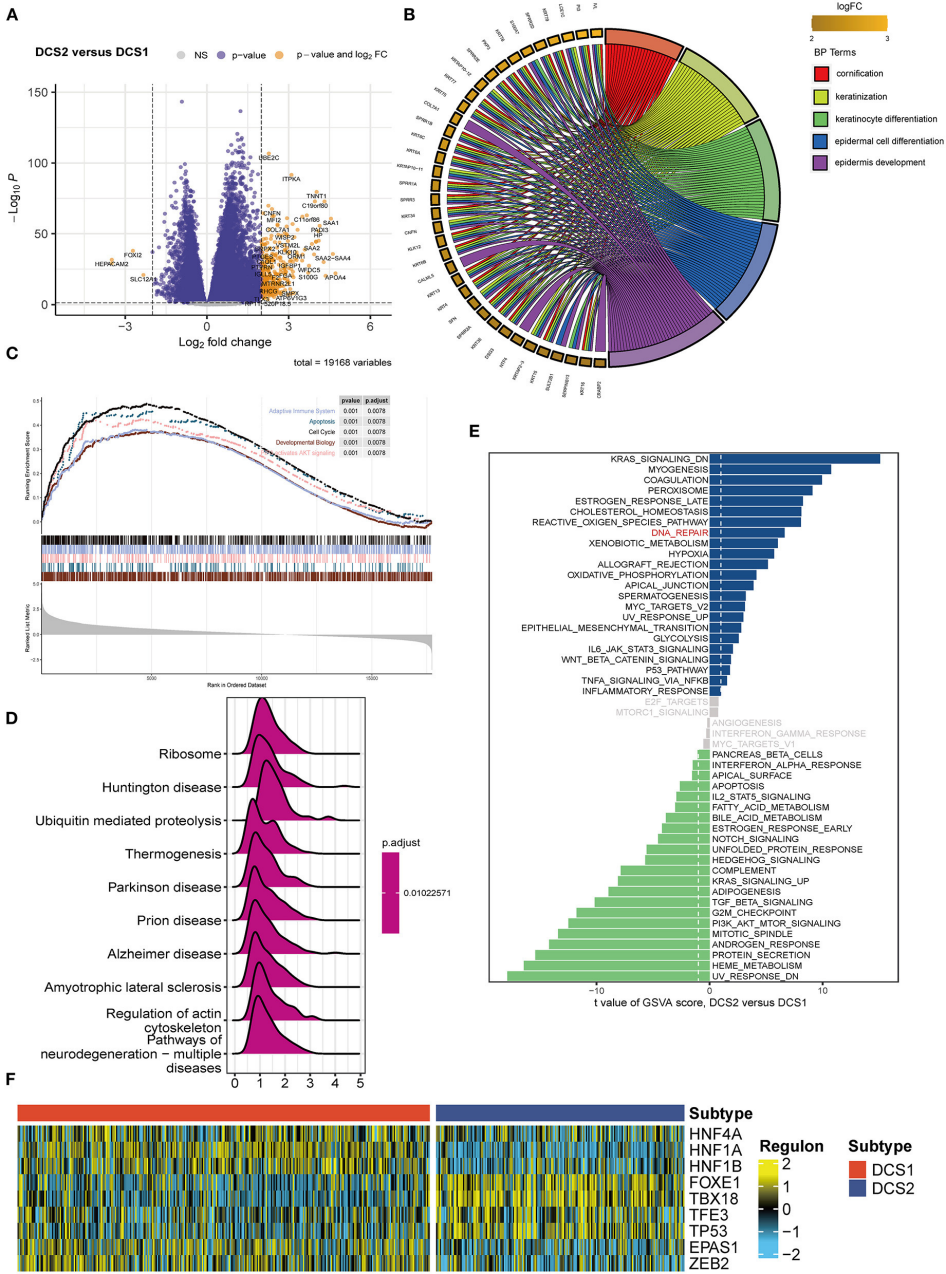
Functional enrichment analysis of ccRCC subtypes. **(A)** Volcano plot showed DEGs. **(B)** BP enrichment analysis, **(C)** GSEA, **(D)** KEGG and **(E)** GSVA analysis between subtypes. **(F)** Different transcriptional factors' regulon scores. Yellow represented activated expression of transcription factors. Blue represented repressed expression of transcription factors.

Since DCS1 and DCS2 led a distinctive prognosis, we next aimed to decipher the biological difference between subtypes. The different expression genes (DEGs) were depicted in Figure 2A. GO enrichment analysis showed that DEGs were mainly involved in cornification, keratinization, and epidermal cell differentiation in BP part; cornified envelope and keratin filament in CC part; and serine-type endopeptidases inhibitor activity and hormone activity in MF parts (Figure 2B, Supplementary Figures S3A,B). We then performed GSEA analysis of the differential genes, which showed that the adaptive immune system, apoptosis, cell cycle, developmental biology and PIP3/AKT signaling pathways were activated in DCS1, whereas DCS2 was in a suppressed state, indicating that it might lead a poor immune response (Figure 2C). KEGG enrichment analysis also showed that it was associated with abnormal protein metabolism, and the differential genes were mainly located in thermogenesis, ribosome, and ubiquitin mediated proteolysis pathways (Figure 2D). To further investigate the differences between genes, we used GSVA analysis to analyze the differences between gene sets. KRAS, myogenesis and coagulation pathways were significantly upregulated in DCS1, while UV_RESPONSE_DN, HEME_METABOLISM and protein secretion pathways were significantly upregulated in DCS2 (Figure 2E). The transcriptome differences were further analyzed by regulon analysis. It was found that HNF4A, HNF1A, HNF1B, EPAS1 and ZEB2 were up-regulated in C1, while FOXE1, TBX18, TFE3 and TP53 were down-regulated in DCS1 (Figure 2F). EPAS1 is a transcription factor that regulates hypoxia-related genes, and its expression increases with the decrease of oxygen concentration (24). It indicates that DCS1 owned a hypoxic state. Tumor hypoxia has been reported to lead to tumor resistance to immunotherapy (25–27), so targeted disruption of the hypoxic environment may make DCS1 more sensitive to immunotherapy. We also compared the metabolism, tumor immune, and classic oncogenic pathways' state difference between DCS1 and DCS2. And found that several pathways were significantly activated in DCS2, including ubiquinone and nucleotide sugar metabolism, sulfur metabolism, linoleic acid metabolism of tumor metabolic pathways; cytokines, chemokines, trafficking of immune cells to tumors, Treg, interleukins, macrophage related signatures and complement of immune part; regulation of exosomal secretion, ferroptosis of tumor related pathways (Supplementary Figures S4A–D).

### Comparing immune infiltration and component of two subgroups

**Figure 3:**
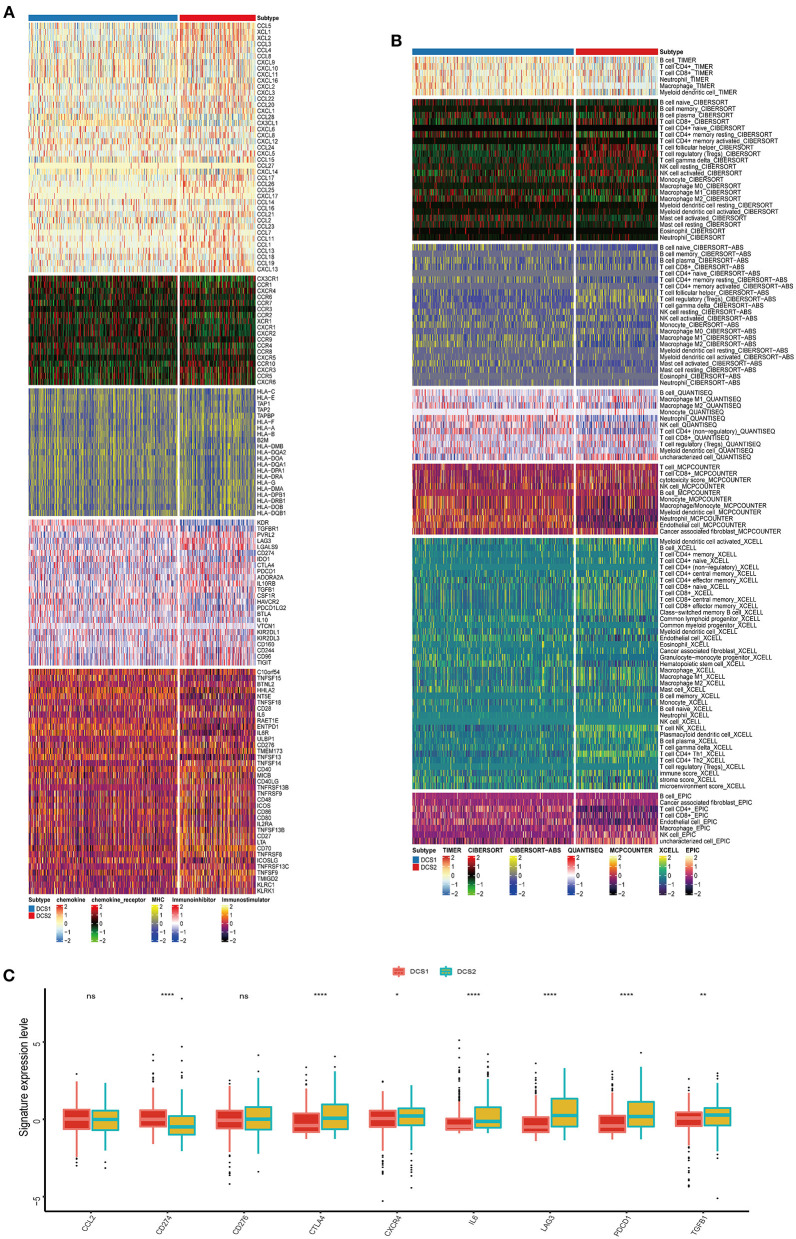
Immune profiling between subtypes. **(A,B)** Heatmap indicating the different immune signatures and immune component enrichment between subtypes. **(C)** Different expression level of immune checkpoint inhibitors between subtypes.

The biological enrichment analysis indicated the significant difference in immune related pathways between DCS1 and DCS2, thus we decided to further compare such immune heterogeneity. We found that chemokine related signatures were high expressed in DCS2, while immune related inhibitor and stimulator factors displayed a heterogenous expression pattern between subtypes, which might be partially accounted for the different DDR related signatures expression pattern (Figure 3A). We used several deconvolution algorithms to describe the immune infiltration of subtypes and analyze the heterogenous composition of TME. The results were consistent and showed that DCS2 displayed lower immune cell infiltration than DCS1 (Figure 3B). Most immune cells were highly infiltrated in DCS1, while neutrophil and endothelial cells were significantly enriched in DCS2.

**Figure 4:**
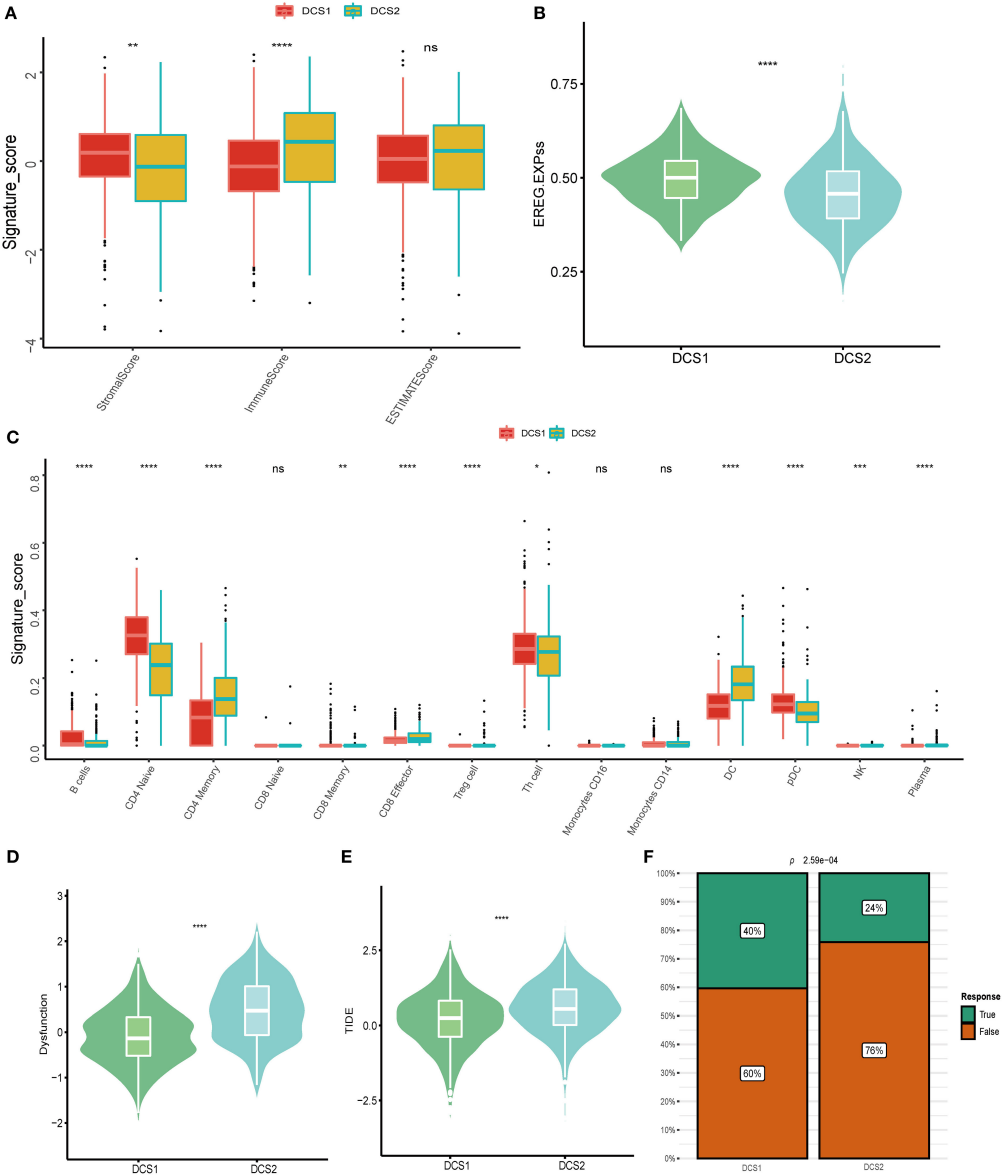
Landscapes of specific immune components and immune function scores. **(A)** Stromal, immune and ESTIMATE scores difference between subtypes. **(B,C)** EGER.EXPss and immune signature difference between DCS1 and DCS2. **(D,E)** Immune dysfunction and TIDE score between subtypes. **(F)** Difference of immune therapy response of DCS1 and DCS2.

Except of CD274, most immune check point, or immune exhausted signature, including CTLA4, CXCR4, IL6, LAG3, PCDC1 and TGFB1, were higher expressed in DCS2 (Figure 3C). All those results reminded that DCS2 might led an immune exhausted phenotype. Through estimate algorithm, we found that stromal score was higher in DCS1, while immune and ESTIMATE scores were higher in DCS2 (Figure 4A). Epigenetically regulated RNA expression-based stemness score (EREG.EXPss) was also higher in DCS1 (Figure 4B). The immune cell infiltration scores calculated *via* TIP pipeline revealed that B cell, CD4 naïve, Th cell, pDC signatures were lower in DCS2 (Figure 4C). Dysfunction and TIDE scores in DCS2 were significantly high in DCS2 (Figures 4D,E). Consistently, the immune therapy response rate in DCS1 were higher than DCS2 (40 vs. 24%) (Figure 4F). Combined with paradox results of clinical outcome and immune infiltration difference, we hypothesized that the DCS2 might be an immune-desert or exhausted state with the mark of suppression in immunity, and such results might be relevant to deregulated DDR pattern between subtypes.

### Genomic mutation of different subtypes

**Figure 5:**
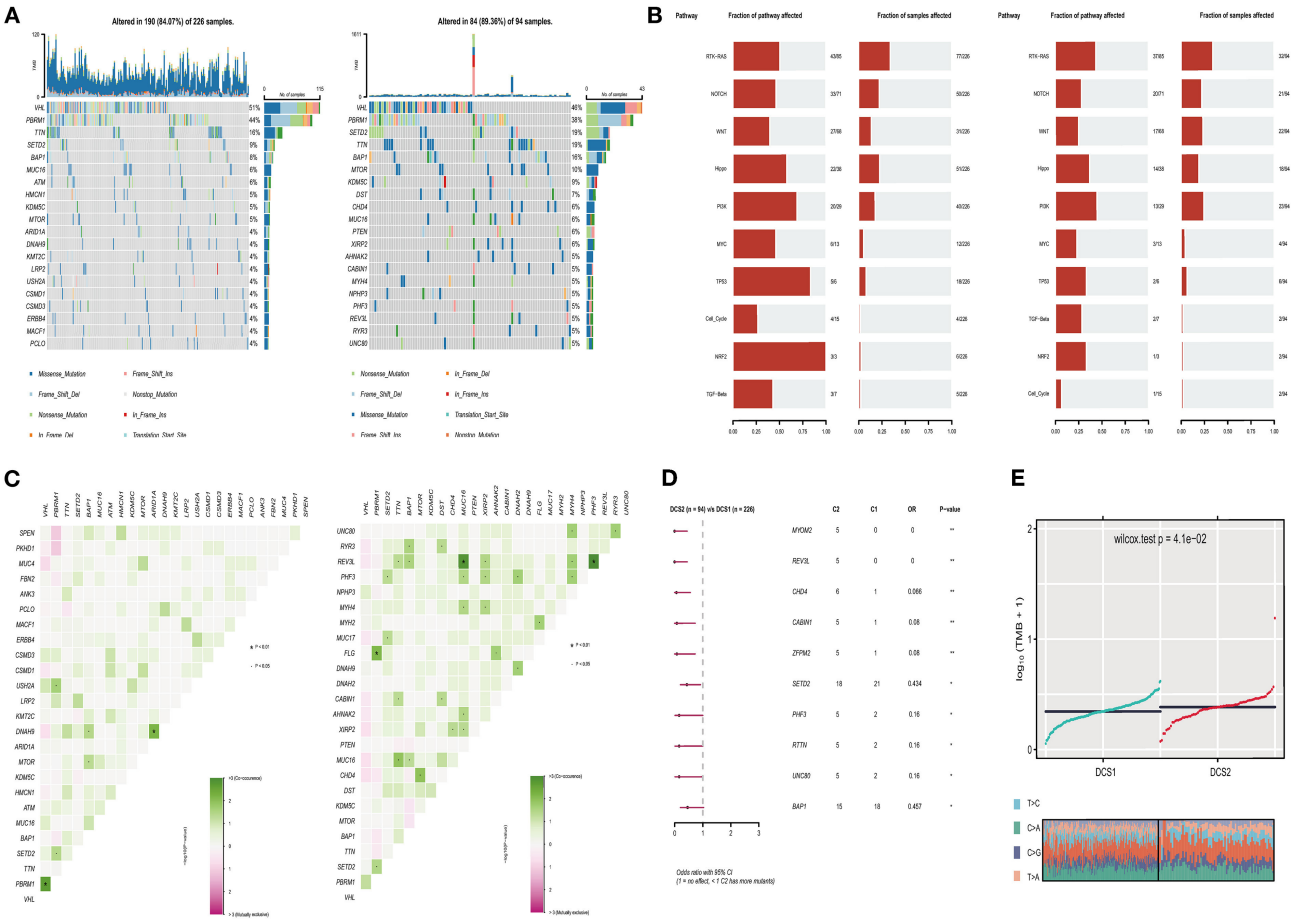
Profiles of somatic mutations between the two subtypes. **(A)** Mutation landscape of DCS1 and DCS2, containing the top 20 mutated signatures. **(B)** Oncogenic signaling pathways in DCS1 and DCS2. **(C)** Co-mutation and -existing mutation pattern in DCS1 and DCS2. **(D)** Forest plot showing prognostic impact of mutated signatures between subtypes. **(E)** Tumor mutation burden difference between subtypes.

The alteration of genome was analyzed to decipher the potential oncogenic factors in DCS1 and DCS2. The most frequent mutation signatures were depicted in Figure 5A. The overall mutation frequency of DCS1 was lower than DCS2 (84.07 vs. 89.36%). When compared with DCS1, DCS2 displayed several high frequencies in signatures, including BAP1, mTOR, KDM5C, DST, CHD4, PTEN and so on (Figure 5A). We also evaluated somatic alterations in common tumor associated pathways in two subgroups, including RTK-RAS, Hippo, WNT, PI3K, NOTCH, MYC, NRF2, TP53, TGF-Beta, and Cell cycle (28). The results showed that NRF2 and TP53 were affected in DCS1, while RTK-Ras and PI3K pathways were most affected in DCS2 (Figure 5B). Interestingly, we found that co-mutation frequency was lower in DCS1, containing ARID1A-DNAH9 (*p* < 0.01); while such patterns in DCS2 contained PBRM1-FLG, MUC16-REV3L, PHF3-REV3L (*p* < 0.01) (Figure 5C). Most mutated signatures led protective roles in DCS2, including MYOM2, REV3L, CHD4, CABIN1, ZFPM2, SETD2, PHF3, RTTN, UNC80 and BAP1 (Figure 5D). Consistently, the average tumor mutation burden was higher in DCS2 (Figure 5E).

**Figure 6:**
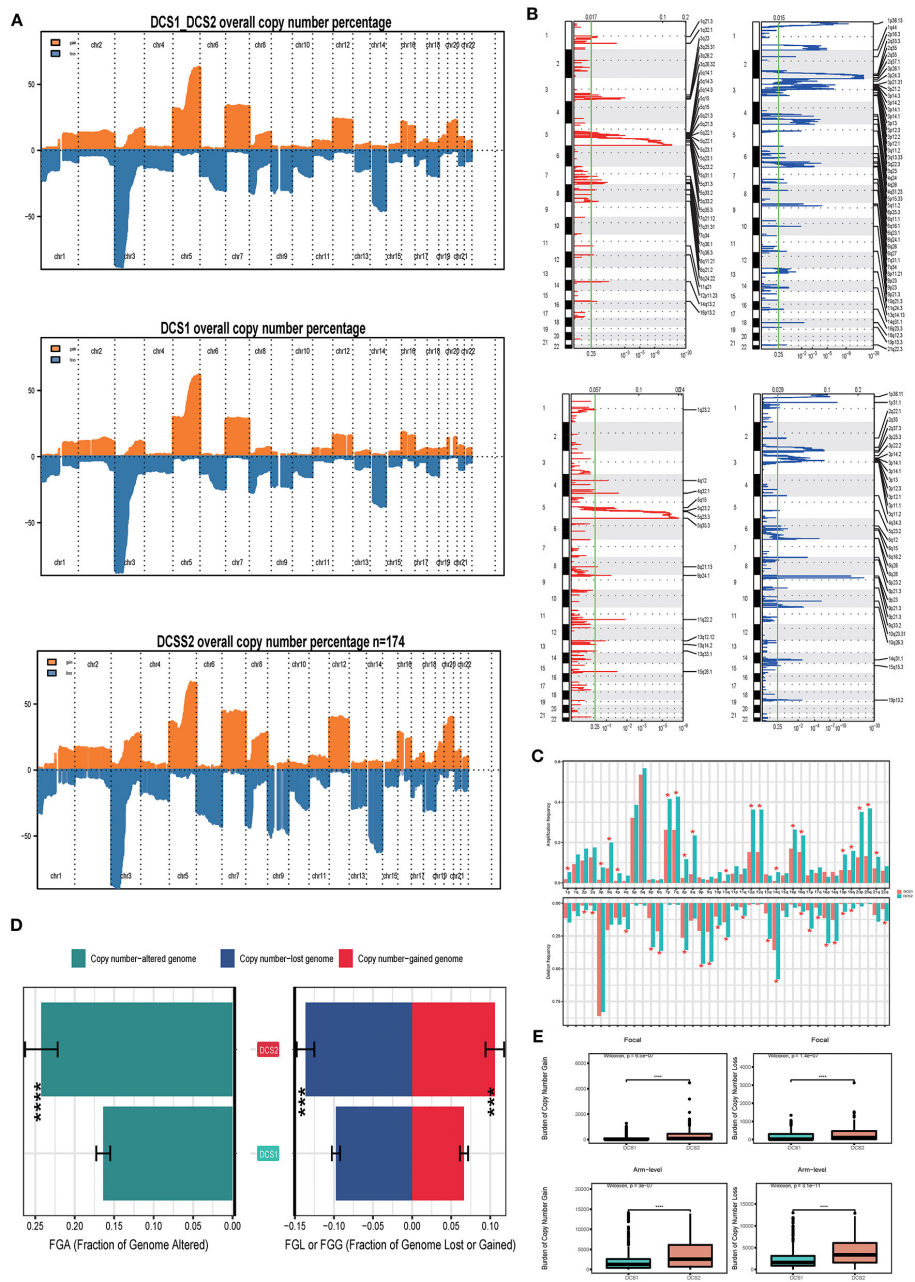
Landscapes of copy number variations. **(A)** Comparison of overall copy number among all patients, DCS1 and DCS2. Orange represents genomic gain; blue represents genomic loss. **(B)** Detailed specific amplification or deletion sites between subtypes. Up represents DCS1; Low represents DCS2. **(C)** The amplification or deletion frequency in chromosome between subtypes. **(D)** Bar-plot indicating total alteration frequency. **(E)** Different burden of copy number gain at focal and arm-level. The *, **, ***, and **** symbols indicate the values of *P* < 0.05, *P* < 0.01, *P* < 0.001, and *P* < 0.0001 respectively.

We also compared the CNV differences between subgroups, and the results showed that the CNV occurrence frequency was higher in DCS2 (Figures 6A–C). In detail, amplification in chr 1p, 3p, 3q, 4p, 7p, 7q, 8p, 8q, 10q, 12p, 12q, 14q, 16p, 16q, 19p, 19q, 20p, 20q and 21q, deletion in chr 2p, 2q, 4q, 6p, 6q, 8p, 9p, 9q, 10p, 10q, 11q, 13q, 14q, 16q, 17p, 17q, 18p, 18q, 19p, 19q and 22q were higher in DCS2. The total copy number alteration rate also proved such difference (Figure 6D). When it mentioned to focal, or arm-level mutation level, DCS2 subtypes displayed a higher rate comparing with DCS1 (Figure 6E).

### Drug sensitivity profiles of different DDR clusters

**Figure 7:**
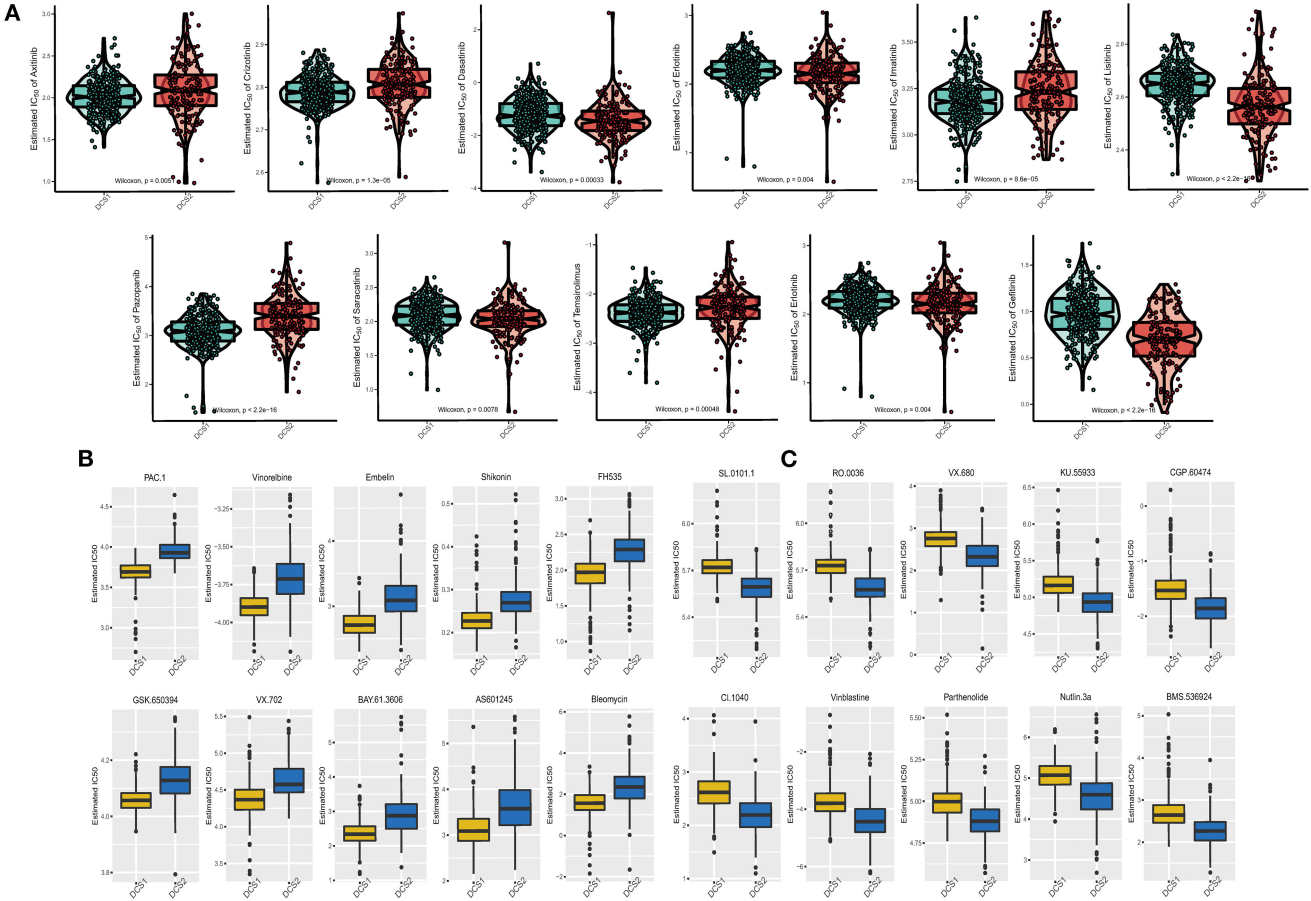
Drug sensitivity difference between DCS1 and DCS2. **(A)** Distribution of IC50 value of clinical chemotherapy agents. **(B,C)** Novel identified molecular agents for DCS1 and DCS2, respectively.

Drug response data (as defined by IC50 values) were collected from the GDSC database to analyze drug sensitivity difference between subtypes. We found that most of the drugs performed poorly in the DCS2 (Figure 7A), which was consistent with previous prognostic results. The IC50 was higher in DCS2 when treated with Axitinib, Crizotinib, Imatinib, Pazopanib, Temsirolimus, while Dasatinib, Erlotinib, Lisitinib, Saracatinib, Erlotinib and Gefitinib might be novel therapeutic targets for such a high-risk subtype. Figures 7B,C showed the top 10 potential drugs with the most significant differences between subgroups. The DCS1 was sensitive to PAC.1, Vinorelbine, and Embelin, while the DCS2 group responded better to SL.0101.1, RO.0036, VX.680, and KU.55933. To further assess the results' reliability and identify novel treatment target, we applied ccRCC cell lines expression and therapeutic response information from CCLE datasets. The DDR related signature expression pattern in ccRCC cell lines was similar with patients from DCS1 and DCS2 (Supplementary Figure S5A). The AUC of DCS1 was higher in FTI-277, KIN001-270, PD-173074 and PAZOPANIB and the AUC of DCS2 was higher in EHT 1864, GEFITINIB, A832234, KOBE2602 and ALBOCICLIB (Supplementary Figure S5B). All those agents might be helpful for precise management of ccRCC patients, and potential.

### Validation of the robustness of subtyping models using external datasets

Even the re-subtype system of DCS1 and DCS2 in TCGA-KIRC cohort received promising results, whether such a classifier could decode the heterogeneity in other datasets remained unknown. We applied NTP algorithm to perform re-cluster analysis in three independent cohorts. Cluster-specific signatures were identified using the nearest template prediction (NTP) algorithm (Supplementary Table S3) from TCGA-ccRCC, which divided the ccRCC patients form TCGA-KIRC, Motzer's and Miao's studies into DCS1 and DCS2 subgroups (Supplementary Figures S6A–C). ccRCC patients re-clustered into DCS2 also owned an inferior prognosis compared with DCS1, which was consistent with previous survival results. All these results confirmed the reliability and robustness of our classification model.

### Construction and validation of subtypes' biomarkers-based risk score

Since the dysregulated DDR expression pattern could led distinctive clinical outcome and multi-omics level-based heterogeneity in ccRCC patients. Thus, we next aimed to develop a novel subtypes' specific biomarkers related risk score system. We firstly identified prognostic related signatures from biomarkers retracted from DCS1 and DCS2, then we ranked those signatures according to their contributor importance on OS (Supplementary Figures S7A,B). Finally, we constructed a DDRsig = 8.34047^*^PLK1- 5.617764^*^SMARCA2-6.195526^*^MSH3 according to RSFVH algorithm. Patients in training dataset, TCGA-KIRC, and test dataset, KIRC-JAPAN, were divided into high-risk and low-risk subgroups when applying median score as the cutoff (Supplementary Figures S7C,D). Comparison of survival probabilities revealed that patients in the high-risk subgroup all had significantly worse prognosis than the low-risk subgroup (Supplementary Figure S7E). Area under the ROC curve was used to evaluate the specificity and sensitivity of the DDRsig score model in both the TCGA-ccRCC and TCGA-JAPAN. AUC scores were above 0.7, which suggested that our model reached a good prognostic prediction (Supplementary Figure S7F). These results indicate that the constructed score was reliable enough to be used to assess the prognosis of ccRCC patients.

### DDX1 functions as the core signature in CcRCC

**Figure 8:**
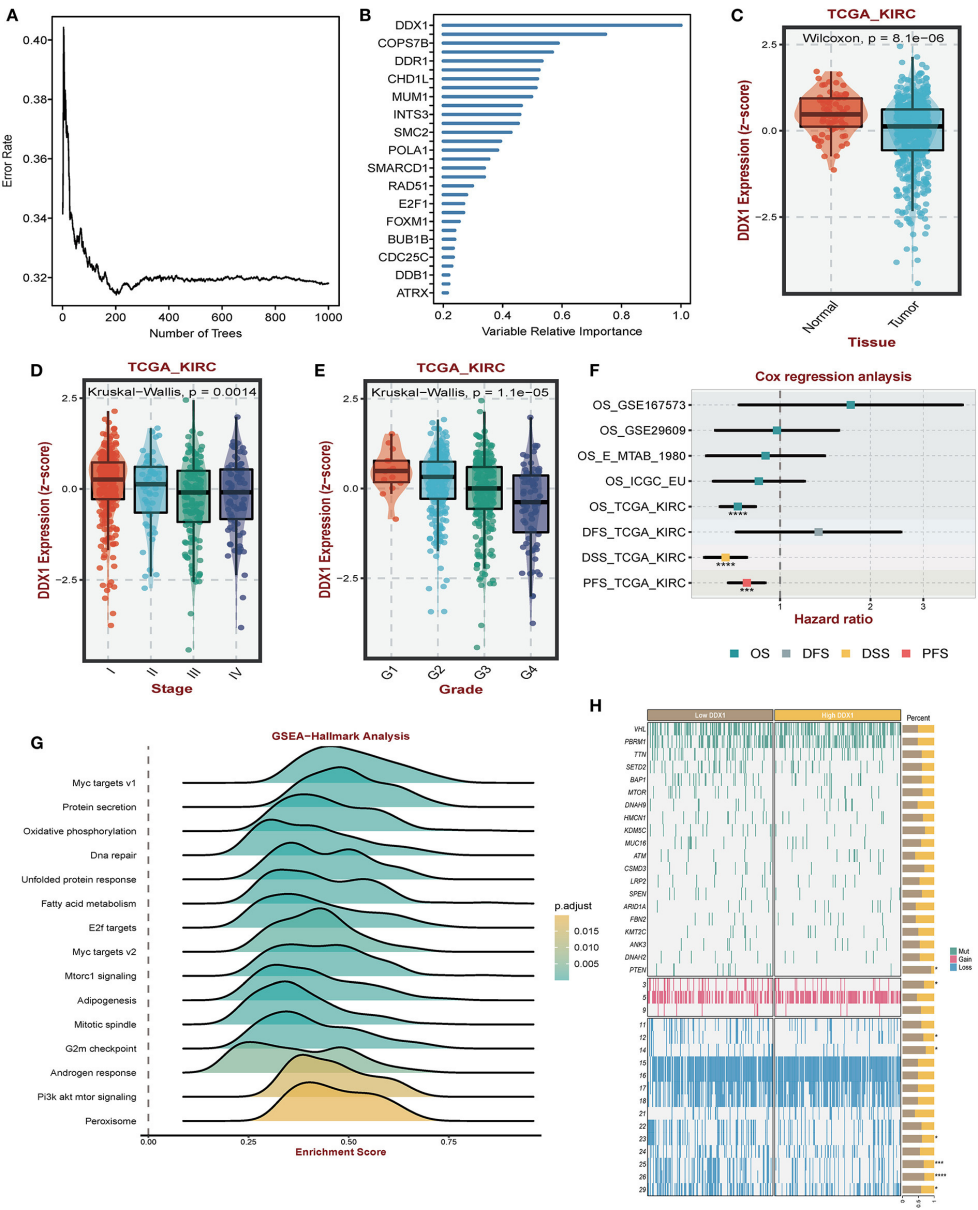
Impact of DDX1 in ccRCC. **(A,B)** Radom Forest tree indicating the importance of DDR-related signatures. **(C)** Different expression level of DDX1 in normal and tumor tissues. **(D,E)** Expression level of DDX1 in different stage and grade tumor tissues. **(F)** Survival impact of DDX1 in ccRCC across different datasets. **(G)** GSEA-hallmark analysis based on DDX1 expression level in ccRCC. **(H)** Mutation and genomic landscape between DDX1^low^ and DD1^low^ groups in ccRCC.

Considering the regulatory role of DDR-related signatures and distinctive prognosis between subtypes, we analyzed which gene was the most important one. Among biomarkers from DCS2, we observed that DDX1 might play a central role in ccRCC patients' prognosis via Random Forest analysis (Figures 8A,B). Comparing with normal tissues, the expression level of DDX1 was significantly de-regulated in tumor tissues (Figure 8C). In addition, we found DDX1 expression level was lower in late stage and grade tumor tissue (Figures 8D,E). Across different ccRCC datasets, we revealed that DDX1 could be treated as a protective factor, especially in TCGA-KIRC (Figure 8F). To further investigate the biological impact in ccRCC, we performed GSEA analysis though ORA algorithms and found that DDX1 was associated with Myc and PI3K-AKT-MTOR signaling (Figure 8G). To explain the aberrant expression of DDX1 in ccRCC, we divided all ccRCC patient to DDX1^low^ and DDX1^high^ according to median expression level; And found that mutation frequency of PTEN, both of gain and loss in chromosome were higher in DDX1^low^ subtype (Figure 8H).

## Discussion

Clear cell renal cell carcinoma (ccRCC) is characterized by extreme high level of heterogeneity, which is one of the reasons for the unsatisfactory results of immunotherapy (29). Therefore, there is an urgent need to distinguish molecular subtypes of ccRCC and to predict patient prognosis and enhance immunotherapeutic response. DNA damage signature and its associated repair mechanisms lead a pivotal role in carcinogenesis, as most oncogenic alterations (including mutations, translocations, amplifications and deletions) in human are derived from inefficient repair of damaged DNA (30). It is involved in all processes from early precancerous lesions to metastasis of tumors and has altered functions, manifesting as tumor suppressor in early stages and as tumor promoter in late tumor stages (31–33). And it has been shown that mutations in DDR are associated with tumor resistance to radiotherapy (34).

The function of DDR pathway is diverse, while the studies based on DDR molecular clustering in ccRCC remain few and unknown. In this study, we analyzed DDR regulatory signatures in multi datasets at multi omics level. We observed that DDR related signatures were significantly upregulated in various cancer tissues compared to paraneoplastic tissues, which were associated with genomic mutations and epigenetic modifications. Based on the expression of DDR related signatures, ccRCC patients can be classified into two different DDR regulatory clusters (DCS1 and DCS2). Between them, the DCS2 cluster has a poor survival probability, which may be related to its higher tumor mutation burden, activated metabolic profile and immunosuppressive status. In addition, the prognostic risk model constructed based on subgroup characteristics achieved good results in both training and validation cohorts; in addition, the predictive accuracy in 1-, 3- and 5-year OS was higher than previous prognostic models (20, 35, 36). Finally, we analyzed the core signature in the DDR gene set and found that DDX1, as a pivotal prognostic factor in the DDR axis, played a good prognostic predictive role and can be a reliable ccRCC target.

Immune checkpoint inhibitors (ICI) combined with tyrosine kinase inhibitors (TKI) have become the first-line treatment for ccRCC. However, only some patients respond well to the therapy, and the objective response rate varies between different drug combinations with the range of 41–71% (37–39). One possible solution is combination therapy, in which DDR modulation targets may cooperate with immunotherapy. Strong evidence suggests that defects in DDR-related signaling pathways cause genetic instability, increase tumor mutational load (TMB), generate more mutation-associated neoantigens (MANAs) that are easily recognized by the immune system, and thus enhance the efficacy of immune checkpoint inhibitors (40, 41). Meanwhile, when DDR is absent, the damaged DNA enters the cytoplasm and activates the CGAS-STING signaling pathway, which is related to the activation of innate immunity and CD8^+^ cytotoxic T cells and mediates tumor immunity (42, 43). Mutations in DDR also induce the expression of some proteins on the cell surface to help tumors evade immune surveillance, such as NKD2D (44). Our previous analysis also identified activation of pathways related to protein secretion and protein targeting to membrane. Therefore, the DDR pathway can inhibit immune response by reducing the production of tumor neoantigens and inhibiting the CGAS-STING signaling pathway, which is consistent with our findings of DCS2 clusters. It expresses lower levels of immune cell infiltration and is marked by suppressive status in immunity.

Although there are multiple types of DNA damage, dMMR (defective DNA mismatch repair) remains the only genomic biomarker proven to respond to ICI (45). In addition to this, novel drugs targeting DNA repair proteins, including PARP inhibitors and inhibitors of ATM, ATM and ATR, as well as CHK1, may also play a role with the combination of ICI. PARP inhibitors have been observed to have immunomodulatory effects in tumors, including upregulation of PD-L1 expression in preclinical models and increased CD8^+^ and NK cell infiltration, suggesting a possible role in combination with ICI (46, 47). Clinical trials of PD-(L)1 inhibitors in combination with PARP inhibitors were underway to further evaluate the activity of such combination. Friedlander et al. conducted a phase I clinical trial with 49 cases of the PD-1 inhibitor tesilizumab in combination with the PARP inhibitor pamiparib for the treatment of advanced cancers with possible DNA damage repair defects. The preliminary results showed an ORR of 20% and a clinical benefit rate of 39% (48). The ongoing phase II MEDIOLA trial is evaluating the efficacy of the PD-L1 inhibitor durvalumab and the PARP inhibitor olaparib in cancers with BRCA1 /2 mutations, with results showing a 12-week DCR of 80% (49). Our results showed that DDR-related signatures were aberrantly upregulated in DCS2 as an immunosuppressive subtype. Combined inhibition of DDR and immune checkpoints may increase tumor genomic instability, reshape the ccRCC microenvironment, and promote drug action by restoring immune homeostasis.

In addition to DNA damage repair, ubiquitination and cell cycle-related genes also play important roles in maintaining genomic stability, cancer development and drug resistance (50). Similarly, our study also found that in addition to immune infiltration, DDR is also involved in protein metabolism, cell cycle and other cancer signaling pathways. DCS2 is associated with ribosome production and protein ubiquitination, which affects the metabolism of protein. Post-translational modifications can identify abnormal translated proteins and degrade them, preventing the accumulation of DNA damage (51, 52). The core pathways of DDR, ATM, ATR and DNA-PKcs, function by phosphorylating proteins and thus generating cascade reactions (53). The ubiquitination modification also plays an important role in genome stabilization (54). Wu et al. found that the deubiquitinating enzyme USP37 can act with the helicase BLM to regulate the DNA damage response (55), and Kim et al. also found that ubiquitin enzyme play an important role in poly (ADP-ribose) (PAR) repair of DNA damage (56). Ubiquitination is also closely related to cancer, among which ubiquitination regulation by tumor inhibitor p53 is one of the classical pathways (57). Xu et al. showed that circPOLR2A regulated UBE3C-mediated ubiquitination and degradation of PEBP1 protein, which then activated the ERK pathway to promote RCC progression (58). DCS2 is also associated with cell cycle genes such as chromosome segregation, and the cell cycle has an important role in genomic stability, which prevents the proliferation of cancer cells in three main ways: (i) stimulating abnormal homologous recombination in the G1 phase of cancer cells; (ii) inducing mitotic mutations in cancer cells; or (iii) deleting cell cycle checkpoint (50). The cell cycle also plays an important role in the development of RCC. Kulkarni et al. found that overexpressed lncRNA TCL6 could inhibit cell proliferation and migration/invasion by interacting with miR-155 and induce cell cycle arrest and apoptosis (59). Li et al. found that restoring the expression of microrNA-99a could induce cell cycle arrest in G1 phase *in vitro* and inhibit the proliferation of RCC (60). In addition, p53 is an important protein that regulates the cell cycle, and it is also involved in the proliferation and metastasis of RCC (61–63).

ccRCC is one of the tumors with a high mutational burden. The biological functions of DDR genes are associated with genomic mutations. The DCS2 subtype has a higher mutation frequency than the DCS1. It retains several high frequency mutated genes, including BAP1, mTOR, and KDM5C. In ccRCC, BAP1 is a key tumor suppressor gene that is involved in some important biological process including DNA repair and transcription in the nucleus, and regulating cell death and mitochondrial metabolism in the cytoplasm, and promotes tumor development when mutated in somatic cells (64). In RCC, BAP1 is the gene with the fourth highest mutation rate and is closely associated with the proliferation and metastasis of RCC (65–67). mTOR gene is also one of the classical mutated genes in RCC, and the drug sirolimus against this target has been approved for the treatment of RCC (68). And a recent study found that in a mouse model, mTOR activation combined with p38MAPK-p53 / p16 axis inactivation can trigger renal cell carcinoma like that in humans, suggesting an important role in tumorigenesis (63). KDM5C, a histone demethylase gene, is involved in regulating a variety of biological processes. Zheng et al. showed that kDM5C mutation promotes ccRCC proliferation by remodeling glycogen metabolism and inhibiting ferroptosis (69). RTK-RAS and PI3K are the most affected oncogenic pathways in DCS2. Both pathways are typical oncogene mutation pathways that play an important role in the development of RCC. The copy number variation was higher in DCS2. Fernandes et al. reported that the most significant copy number alterations in ccRCC were loss of 3p (87.3%), 14q (35.8%) and 6q (29.3%) and increase in 5q (59.7%), 7p (29.3%) and 16q (20.6%). There were 19 related genes localized to important regions of CNA, including SETD2, BAP1, FLT4, PTEN, FGFR4, and NSD1 (70), which is consistent with our findings. Thus, DDR-related genes are involved in tumor heterogeneity through crosstalk with genomic mutations.

As mentioned previously, DDR-related genes affect the efficacy of antitumor drugs. Different subtypes of ccRCC patients have different sensitivity to drugs, which may provide some guidance for clinical treatment. We identified several potential molecular inhibitors for DCS2 subtypes which is insensitivity to many drugs. Ribosomal S6 Kinase (RSK) inhibitor SL.0101.1, aurora kinase inhibitor VX.680, and inhibitor KU.55933, which targets ATM Kinase, the core pathway of DDR, were more effective in the treatment of DCS2.

We constructed a risk score model to predict prognosis, which included three key differential genes, PLK1, SMARCA2 and MSH3. PLK1 plays a key role in mitosis, and then affects cell proliferation, which is closely related to the occurrence of a variety of cancers. A variety of PLK1 inhibitors have entered clinical trials (71). Chong found that SMARCA2 could regulate the activity of multiple myeloma by interacting with NSD2 (72). MSH3 is closely related to the occurrence and development of colorectal cancer (73–75). Interestingly, we found that DDX1 may play a central role in DDR axis signaling. It is an ATP-dependent RNA helicase (76). Han et al. found that CircLONP2 can recruit DiGeorge syndrome critical region gene 8 (DGCR8) and Drosha complex through DDX1. They interacted with microRNA-17 (pri-miR-17) and promotes its processing, which enhanced colon cancer aggressiveness (77).

Although our study clarified the characteristics of DDR regulators in ccRCC, there are still some limitations. Most of our findings are based on comprehensive bioinformatics analysis, and further experiments are needed to verify the upstream and downstream molecules and related pathways regulated by DDR. In addition, the prediction model may be influenced by some confounding factors, such as race and region. More independent datasets are needed to validate our risk model.

## Conclusion

In summary, we identified two molecular clusters of ccRCC based on DDR and comprehensively explored the role of DDR-regulated signatures in RCC. Under certain conditions, inhibition of DDR-related genes may become an appropriate cancer treatment. By increasing the instability of tumor genome, increasing the exposure of tumor-associated antigens, and activating immune-related pathways, the tumor immune microenvironment can be reshaping to enhance the efficacy of ICI and reduce the occurrence of drug resistance. Our study contributes to a better understanding of the relationship between DDR and ccRCC and provides clinical guidance for the management of ccRCC.

## Data availability statement

The original contributions presented in the study are included in the article/Supplementary material, further inquiries can be directed to the corresponding authors.

## Author contributions

LW, PL, and LQ conceptualized and designed this study. ZWa, BL, and ZWu wrote the first draft of the manuscript. AJ, JS, XF, and YF have contributed equally to this work. All authors contributed to the article and approved the submitted version.

## Funding

This work was supported by National Natural Science Foundation of China [Grant Nos. 81902560, 81730073, and 81872074].

## Conflict of interest

The authors declare that the research was conducted in the absence of any commercial or financial relationships that could be construed as a potential conflict of interest.

## Publisher's note

All claims expressed in this article are solely those of the authors and do not necessarily represent those of their affiliated organizations, or those of the publisher, the editors and the reviewers. Any product that may be evaluated in this article, or claim that may be made by its manufacturer, is not guaranteed or endorsed by the publisher.

## Supplementary material

Supplementary Figure S1 Workflow of this study.

Supplementary Figure S2 Expression level of DDR related regulators between DCS1, DCS2 and normal tissues in ccRCC.

Supplementary Figure S3 **(A)** CC and **(B)** MF function enrichment between subtypes.

Supplementary Figure S4 **(A)** Metabolism, **(B)** Immune, **(C)** Carcinogenic, and **(D)** tumor microenvironment related signature between subtypes.

Supplementary Figure S5 **(A)** Heatmap of DDR related regulators expression level in ccRCC cell lines, which were divide into cluster1 and cluster2. **(B)** Drug sensitivity analysis in the form of normalized AUC value.

Supplementary Figure S6 Verification of remodeling system in **(A)** JAPAN-KIRC **(B)** Cancer cell and **(C)** Miao's studies based on NTP algorithm.

Supplementary Figure S7 Establishment of a novel risk model based on subtypes' biomarkers. **(A)** Volcano plot illustrating the prognostic impact of biomarkers. **(B)** Random forest ranking the importance of top 10 signatures. **(C,D)** Risk score analysis in TCGA-ccRCC and JAPAN-KIRC cohorts. **(E)** Survival analysis of risk score in TCGA-ccRCC (left) and JAPAN-KIRC (right) cohorts. **(G,H)** The time-dependent ROC curves of risk score in TCGA-ccRCC (left) and JAPAN-KIRC (right) cohorts.

Supplementary Table S1 List of DDR related regulators.

Supplementary Table S2 Difference of clinical characteristics of different subtypes.

Supplementary Table S3 Biomarkers of DCS1 and DCS2.
