# Supplementary figures and images for: A novel thinking: DDR axis refines the classification of ccRCC with distinctive prognosis, multi omics landscape and management strategy
Source: Front Public Health. 2022 Nov 21;10:1029509. doi: 10.3389/fpubh.2022.1029509 (PMC9720257; doi:10.3389/fpubh.2022.1029509)

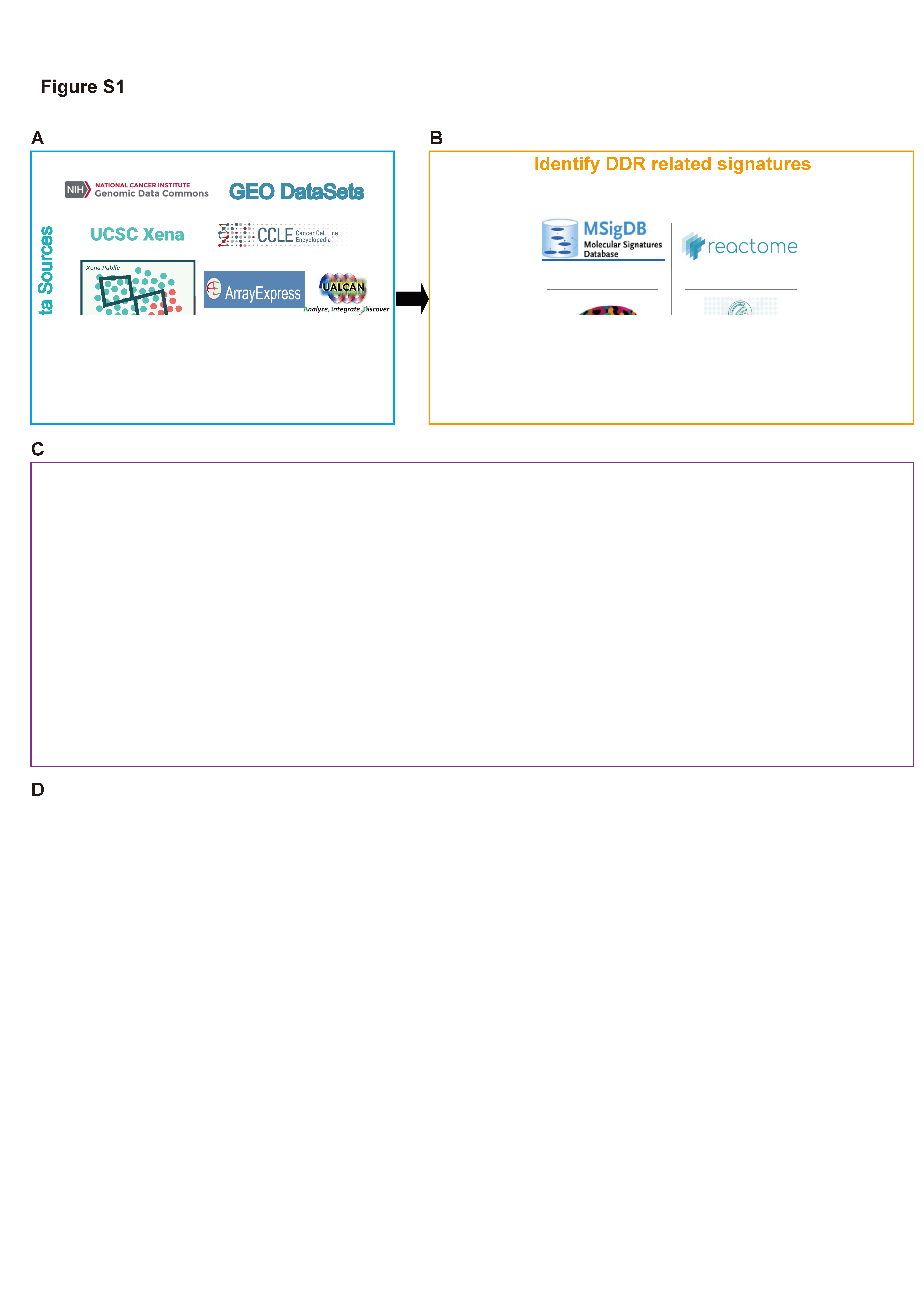

Supplement: Supplementary Figure S1 — Workflow of this study. [file Image_1.TIF]

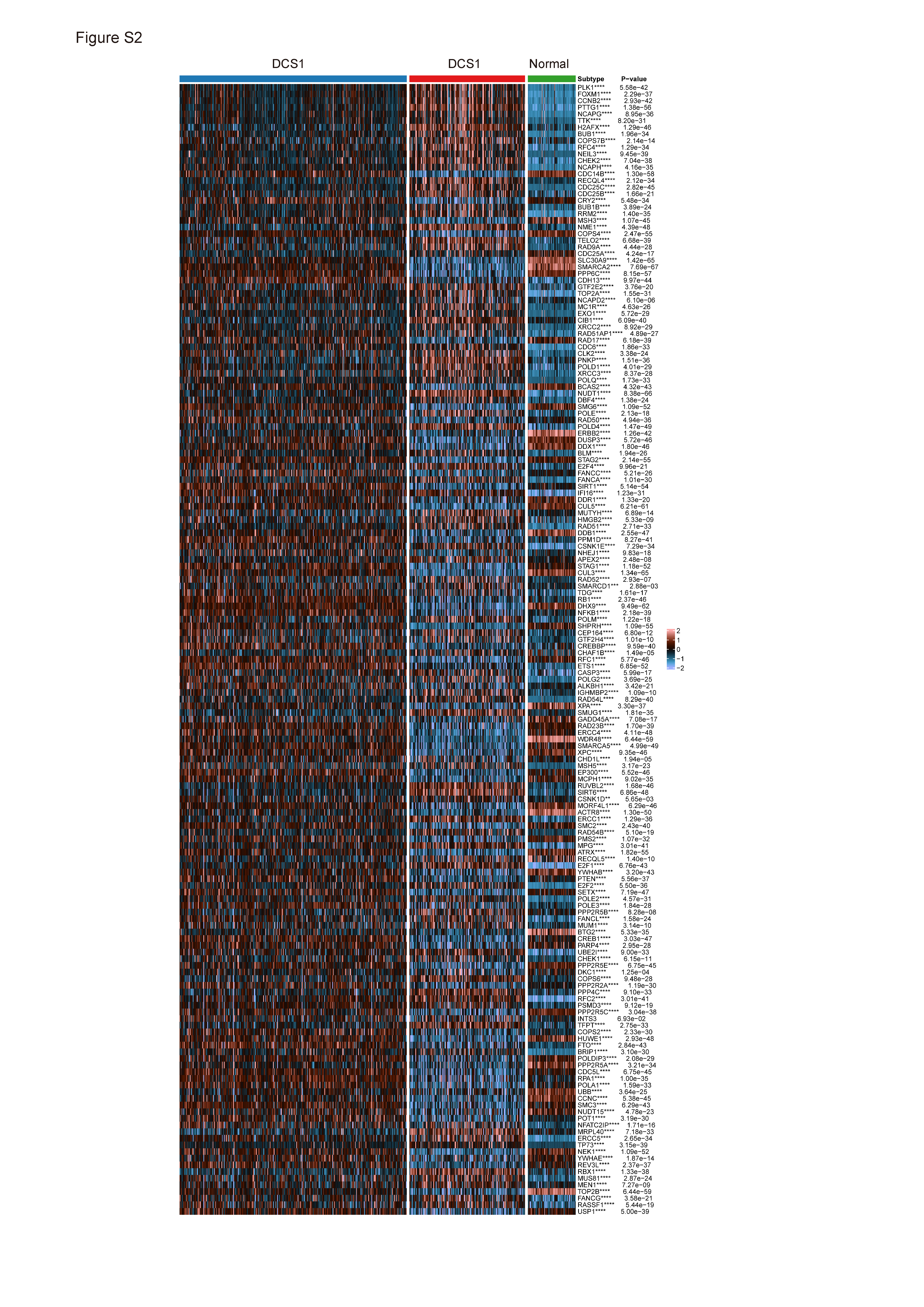

Supplement: Supplementary Figure S2 — Expression level of DDR related regulators between DCS1, DCS2 and normal tissues in ccRCC. [file Image_2.TIF]

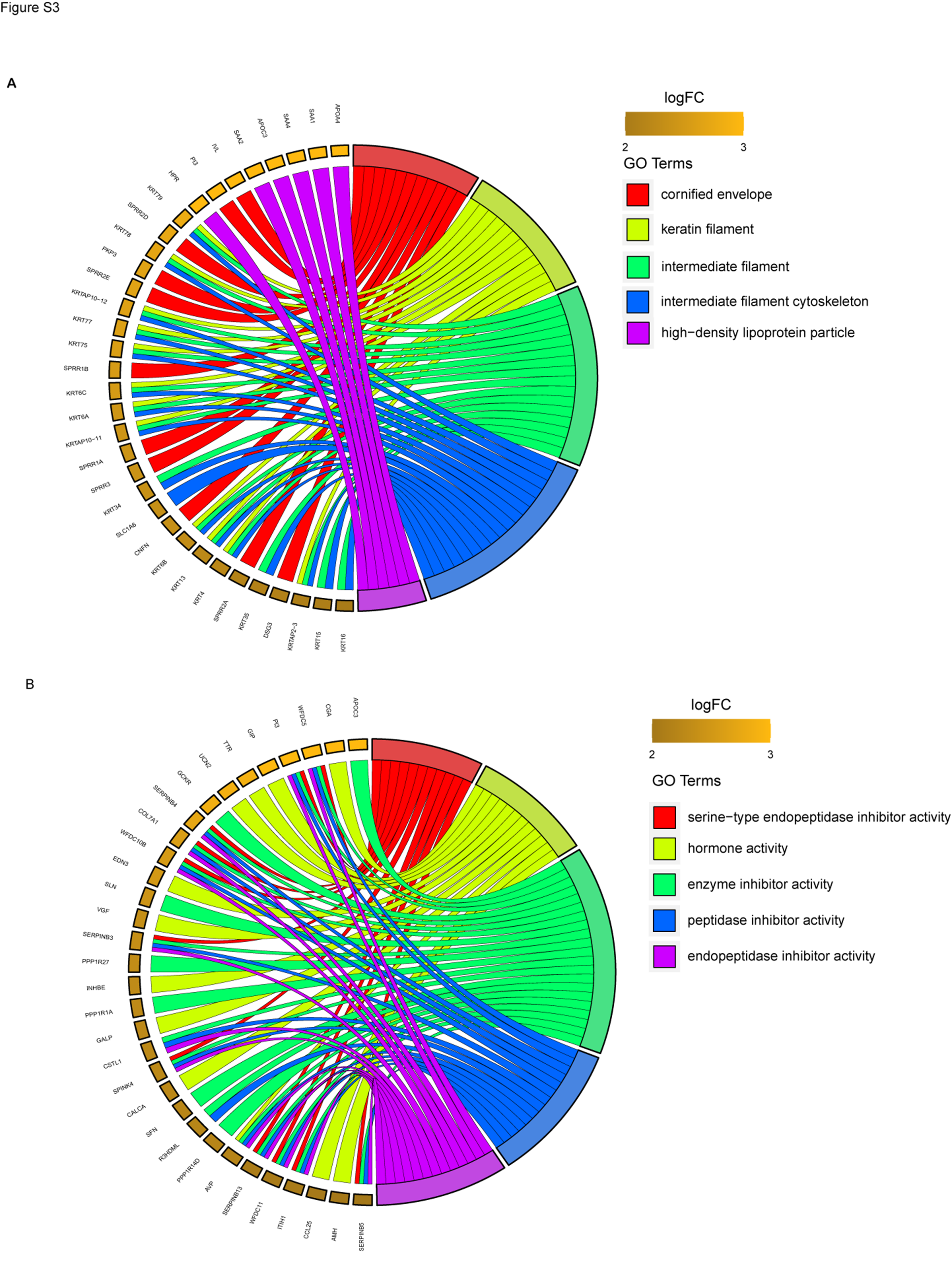

Supplement: Supplementary Figure S3 — (A) CC and (B) MF function enrichment between subtypes. [file Image_3.TIF]

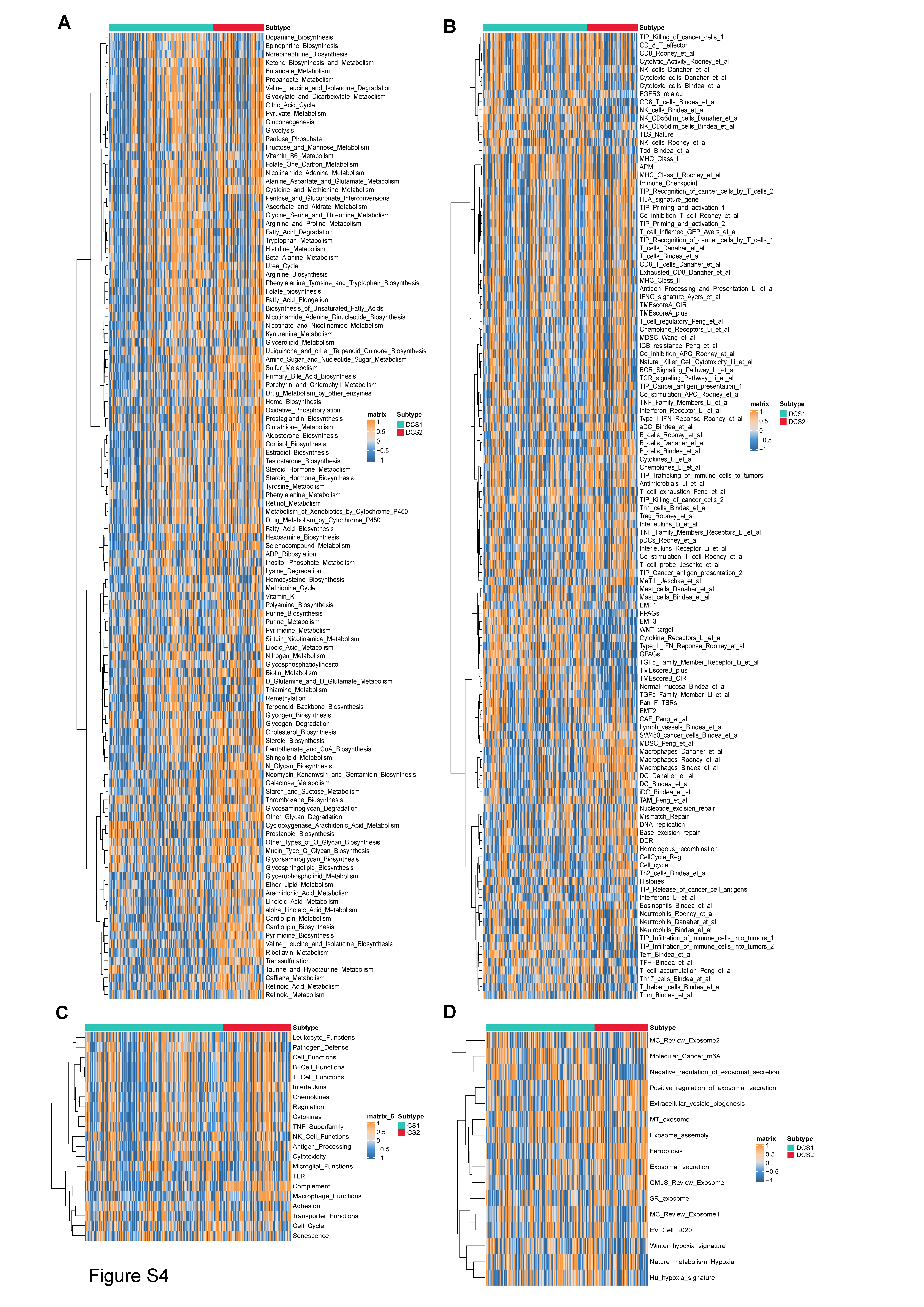

Supplement: Supplementary Figure S4 — (A) Metabolism, (B) Immune, (C) Carcinogenic, and (D) tumor microenvironment related signature between subtypes. [file Image_4.TIF]

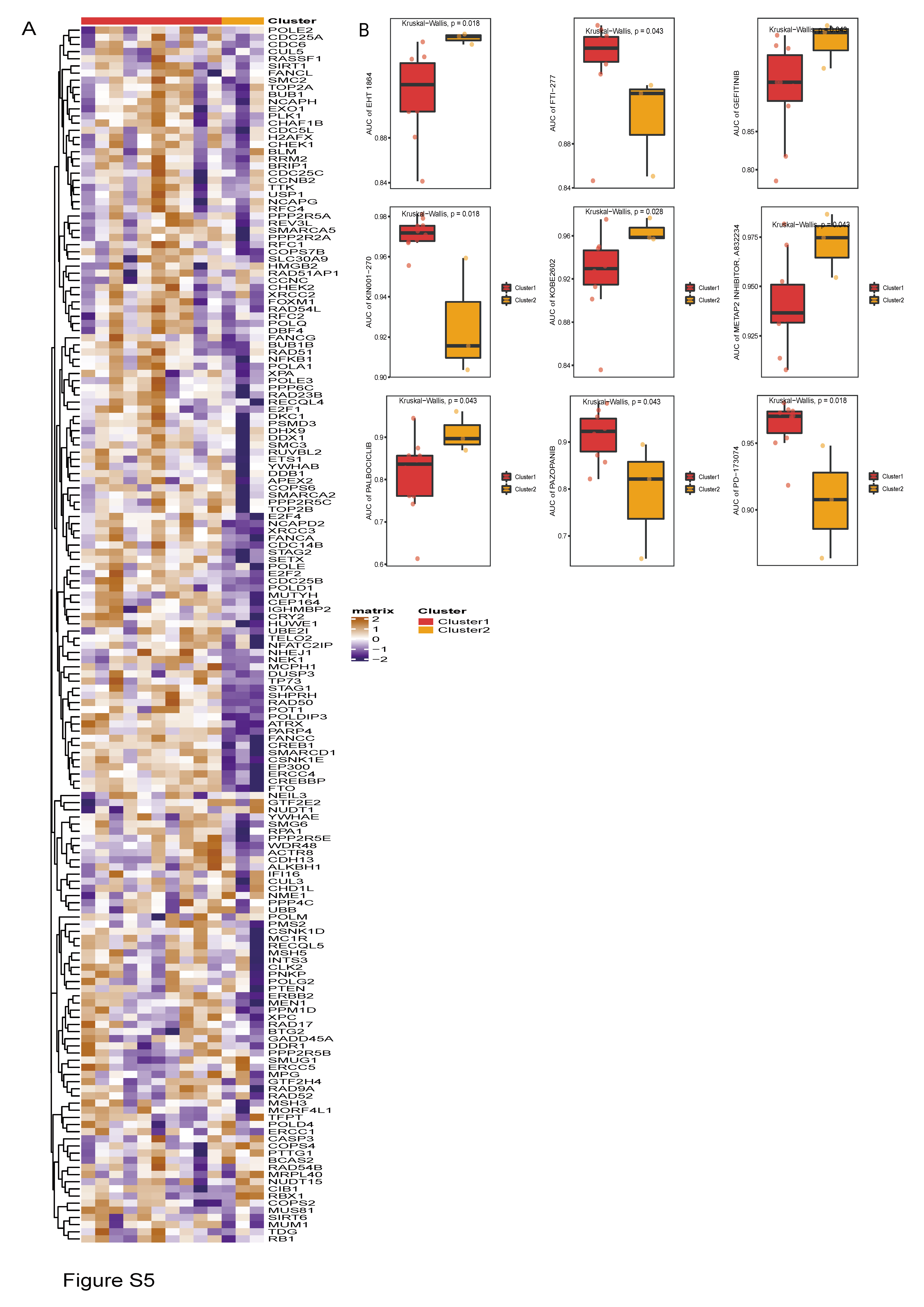

Supplement: Supplementary Figure S5 — (A) Heatmap of DDR related regulators expression level in ccRCC cell lines, which were divide into cluster1 and cluster2. (B) Drug sensitivity analysis in the form of normalized AUC value. [file Image_5.TIF]

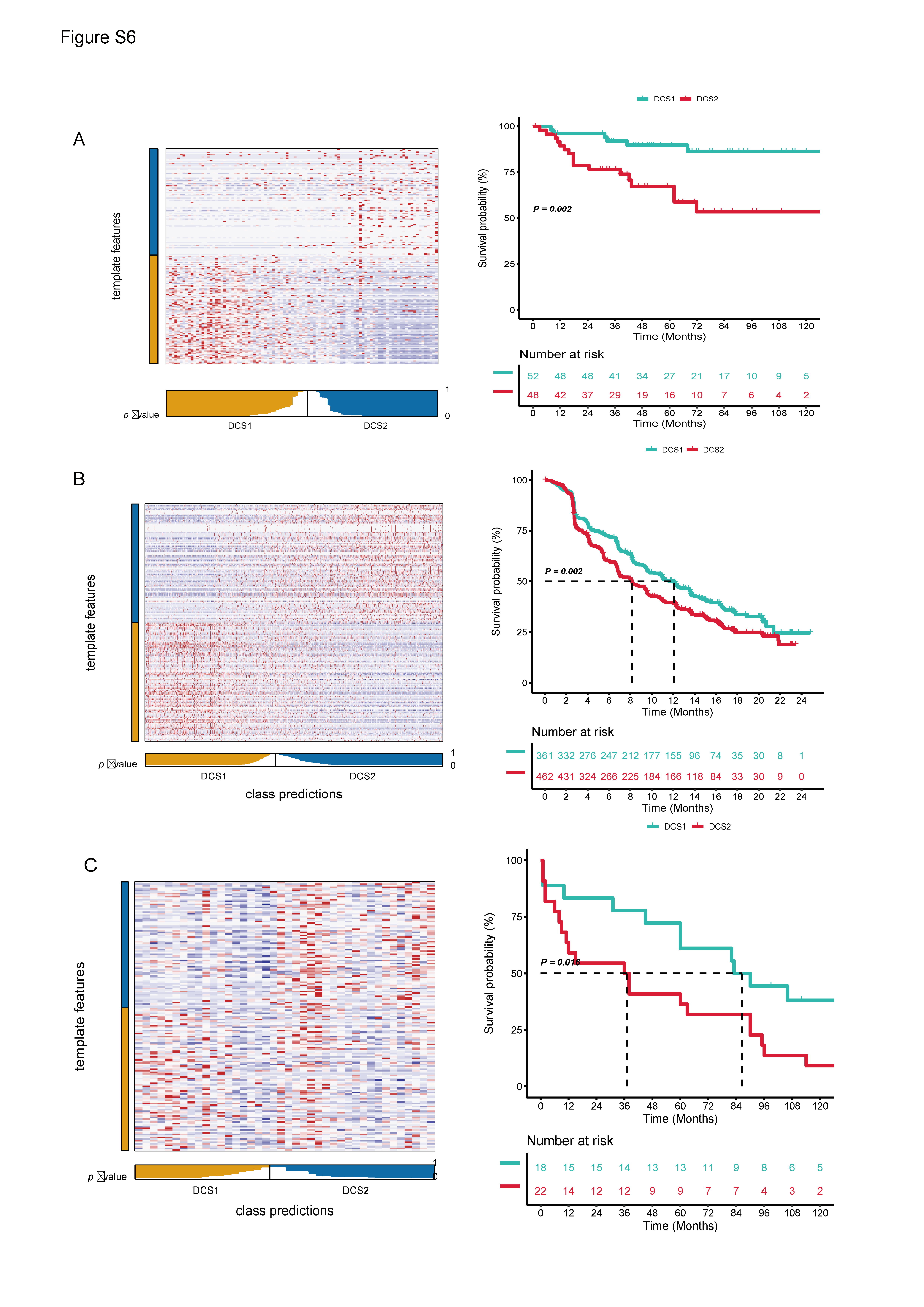

Supplement: Supplementary Figure S6 — Verification of remodeling system in (A) JAPAN-KIRC (B) Cancer cell and (C) Miao's studies based on NTP algorithm. [file Image_6.TIF]

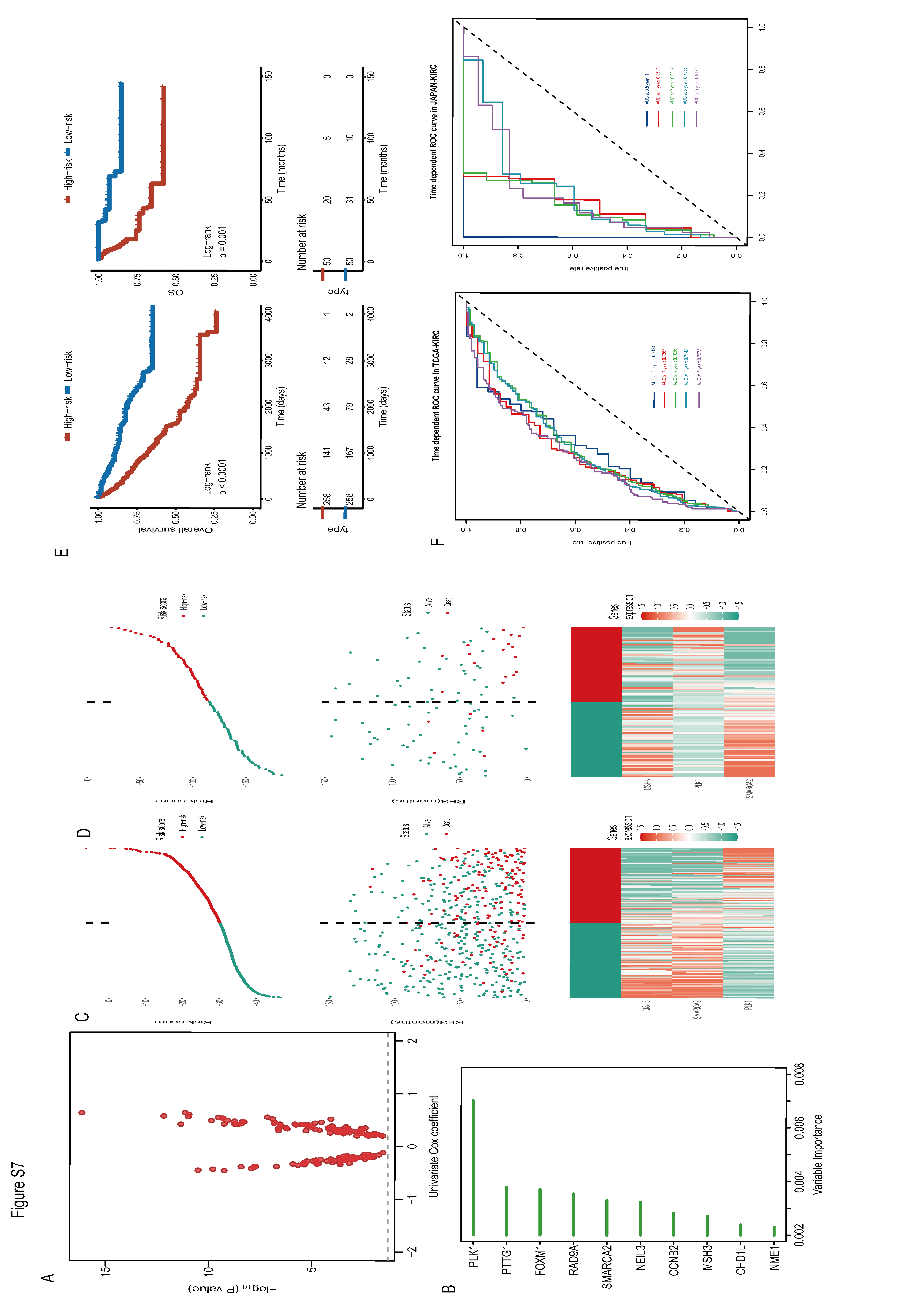

Supplement: Supplementary Figure S7 — Establishment of a novel risk model based on subtypes' biomarkers. (A) Volcano plot illustrating the prognostic impact of biomarkers. (B) Random forest ranking the importance of top 10 signatures. (C,D) Risk score analysis in TCGA-ccRCC and JAPAN-KIRC cohorts. (E) Survival analysis of risk score in TCGA-ccRCC (left) and JAPAN-KIRC (right) cohorts. (G,H) The time-dependent ROC curves of risk score in TCGA-ccRCC (left) and JAPAN-KIRC (right) cohorts. [file Image_7.TIF]
